# Supplementary material for: Digital multiplex ligation assay for highly multiplexed screening of β-lactamase-encoding genes in bacterial isolates
Source: Commun Biol. 2020 May 25;3:264. doi: 10.1038/s42003-020-0980-7 (PMC7248093; doi:10.1038/s42003-020-0980-7)
Supplement: Supplementary file 1 — Supplementary Information [file 42003_2020_980_MOESM1_ESM.pdf]

**Supplementary Material:** Digital Multiplex Ligation Assay for Highly Multiplexed Screening of β-Lactamase-encoding Genes in Bacterial Isolates

**Supplementary Figure 1:** Barchart visualization of the molecular counts for each resistance gene type (y-axis) within each bacterial isolate (x-axis). Limits of detection for each resistance gene type are marked with grey horizontal lines. Colors indicate molecular counts above the detection limit – green if both replicates were above the detection limit and red if only one replicate was above the detection limit.

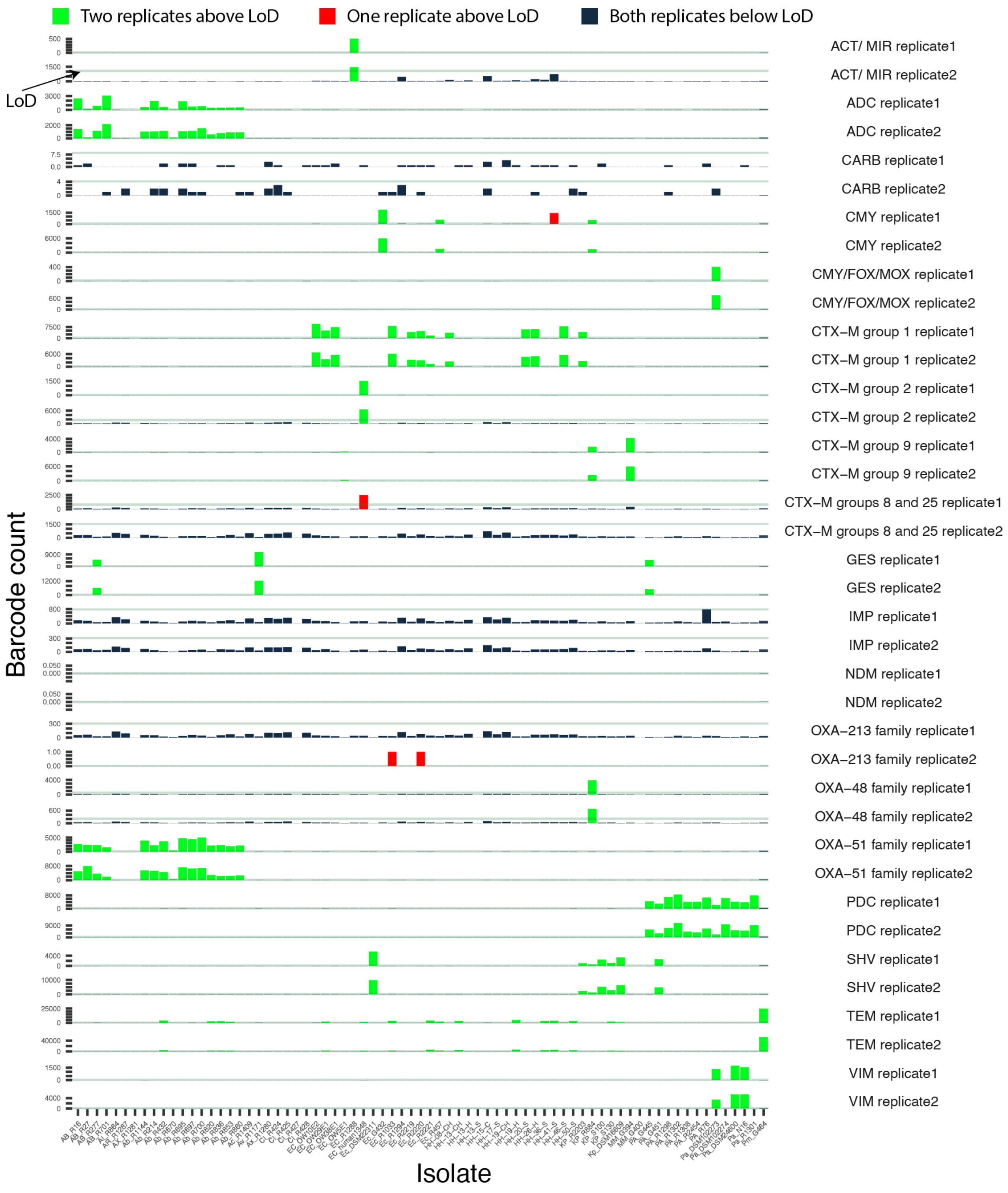

Supplementary Table 1: Probe pair sequences for the 18 clusters covered by the dMLA method, the genes targeted by each probe pair sequence, and associated enzyme family, enzyme class, and distinctive substrates. The sequences cover 1187 out of 1557 (76%) of the β-lactamase genes in the Lahey database. The included targets were chosen based on a combination of clinical relevance and the number of ESBL sequences they target.

| Cluster | No. of genes | Enzyme family | Class | Genes targeted | Distinctive substrates | Probe Set | Oligo Name | Sequence(5'-3') | Length (bp) | 5' Mod |
|---|---|---|---|---|---|---|---|---|---|---|
| 0 | 164 | TEM | A | TEM-1-6/8/10-12/15-17/19-22/24/ 26/28-30/32/35/40/43/45/47-49/52-55/57/60-63/67/68/70-72/76-79/80-88/90-99/10/102/104/116/120-139/ 141-160/162-164/166-169/171/176-178/181-190/193/195/197/198/ 201/205-209-217/219/220/224 | Penicillins, early cephalosporins, extended-spectrum cephalosporins | 0_A_L | C0_1b_oh2_L | TGGGCCCAATTTTCCGTGACAATTAATTCCCTTWTTCCCTTTYTTGCG | 48 | |
| | | | | | | 0_A_R | C0_1b_oh2_R | GCWTTTTGCVTTCCTGTTTTNNNNNNNNNNGAATGAGTGTGCGTGCACTC | 50 | Phos. |
| | | | | | | 0_B_L | C0_3b_oh2_L | TGGGCCCAATTTTCCGTGACAATTAATTTAAGCCCTCCCGTATCGTAK | 48 | |
| | | | | | | 0_B_R | C0_3b_oh2_R | TTATBTACAYGACGGKGRGTNNNNNNNNNNGAATGAGTGTGCGTGCACTC | 50 | Phos. |
| 1 | 159 | OXA (OXA-51 family) | D | OXA-51/64-71/75-80/82-84/86-95/98-100/106-113/115/117/120-128/130-132/138/144/148-150/172-180/194-197/200-203/206/208/216/ 217/219/223/241/242/248/249/250/254/259-263/312-317/336-346/365/ 371/374-391/400-404/406-409/411-414/424-426/429-433/441/442/480/ 497/507-509/516 | Carbapenems | 1_A_L | C1_2b_oh2_L | TGGGCCCAATTTTCCGTGACAATTAATTATCARGATTTASCTCGTCGT | 48 | |
| | | | | | | 1_A_R | C1_2b_oh2_R | ATTGGRCTTGARCTYATGTCNNNNNNNNNNGAATGAGTGTGCGTGCACTC | 50 | Phos. |
| | | | | | | 1_B_L | C1_3b_oh2_L | TGGGCCCAATTTTCCGTGACAATTAATTCCVACAAGTRGGYTGGTTAA | 48 | |
| | | | | | | 1_B_R | C1_3b_oh2_R | CTGRATSGRTTGTTMRGCCTNNNNNNNNNNGAATGAGTGTGCGTGCACTC | 50 | Phos. |
| 2 | 142 | SHV | A | SHV-1/2/2A/3/5/7-9/11/13-16/18/24-29/30-38/40-46/48/49-52/55-57/59-67/69-82/85/86/89/92-111/119-121/128/129/132-135/137/ 141-165/168/172/173/178-180/182/ 183/185-191/193 | Penicillins, early cephalosporins, extended-spectrum cephalosporins | 2_A_L | C2_2_oh2_L | TGGGCCCAATTTTCCGTGACAATTAATTGCCGYCATTACCRTGAGCGA | 48 | |
| | | | | | | 2_A_R | C2_2_oh2_R | TAACAGCGYCGCCAATYTGCNNNNNNNNNNGAATGAGTGTGCGTGCACTC | 50 | Phos. |
| | | | | | | 2_B_L | C2_3_oh2_L | TGGGCCCAATTTTCCGTGACAATTAATTCTGYCGGCGGGCTGGTTTAT | 48 | |
| | | | | | | 2_B_R | C2_3_oh2_R | CGCCGATARGRCCGGAGCTRNNNNNNNNNNGAATGAGTGTGCGTGCACTC | 50 | Phos. |
| 3 | 124 | CMY | C | CFE-1, CMY-2/4-7/12-18/20-51/53-58/60-90/93-97/99-119/121/122/124-135/138/139 and LAT-1/3 | Cephalosporins (CMY-30/37/42 have extended-spectrum) | 3_A_L | C3_1b_oh2_L | TGGGCCCAATTTTCCGTGACAATTAATTAGTCACKCARCAWACKCTGT | 48 | |
| | | | | | | 3_A_R | C3_1b_oh2_R | TTGARYTMGGNTCGGTYAGTNNNNNNNNNNGAATGAGTGTGCGTGCACTC | 50 | Phos. |
| | | | | | | 3_B_L | C3_2b_oh2_L | TGGGCCCAATTTTCCGTGACAATTAATTGCRCTAARMGWYTTTAYKCT | 48 | |
| | | | | | | 3_B_R | C3_2b_oh2_R | AACTCCAGCATTGGTCTKTTNNNNNNNNNNGAATGAGTGTGCGTGCACTC | 50 | Phos. |
| 4 | 72 | PDC | C | PDC-1-18/19a/19b/20/21a/21b/22-26/28/30-32/34-40/43-61/63-72/94-97 | Cephalosporins (PDC-1-5have extended-spectrum) | 4_A_L | C4_1_oh2_L | TGGGCCCAATTTTCCGTGACAATTAATTCCATCAGCCTGAAAGGARAA | 48 | |
| | | | | | | 4_A_R | C4_1_oh2_R | CCGCATTACTTCAGCTATGGNNNNNNNNNNGAATGAGTGTGCGTGCACTC | 50 | Phos. |
| | | | | | | 4_B_L | C4_2_oh2_L | TGGGCCCAATTTTCCGTGACAATTAATTCARCCTGCTCGACCTCGCGA | 48 | |
| | | | | | | 4_B_R | C4_2_oh2_R | CCTATACCGCCGGCGGCTTGNNNNNNNNNNGAATGAGTGTGCGTGCACTC | 50 | Phos. |
| 5 | 68 | CTX-M group 1 | A | CTX-M-1/3/10/12/15/22/23/28-30/32-37/42/52-55/58/60-62/64/ 66/68-72/79/80/82/88/96/101/103/ 114/116/117/123/132/136/139/142/144/150/155-158/162-164/166/167/ | Extended-spectrum cephalosporins | 5_A_L | C5_1_oh2_L | TGGGCCCAATTTTCCGTGACAATTAATTAGTTCACGCTSATGGCGACG | 48 | |
| | | | | | | 5_A_R | C5_1_oh2_R | GCARCCGTCACGCTGTTRTTNNNNNNNNNNGAATGAGTGTGCGTGCACTC | 50 | Phos. |
| | | | | | | 5_B_L | C5_3_oh2_L | TGGGCCCAATTTTCCGTGACAATTAATTCCACCAAYGATATCGCGGTG | 48 | |
| | | | | | | 5_B_R | C5_3_oh2_R | ATYTGGCCAAAAGATCGTGCNNNNNNNNNNGAATGAGTGTGCGTGCACTC | 50 | Phos. |
| 6 | 54 | ADC | C | ADC-1-7/10-23/25/26/30/38/39/41-44/50-54/56/58-64/67/68/73-81 | Cephalosporins, extended-spectrum cephalosporins | 6_A_L | C6_1_oh2_L | TGGGCCCAATTTTCCGTGACAATTAATTTATRATGTRCCNGGTATGGC | 48 | |
| | | | | | | 6_A_R | C6_1_oh2_R | YGTGGGBGTYATTCARAATANNNNNNNNNNGAATGAGTGTGCGTGCACTC | 50 | Phos. |
| | | | | | | 6_B_L | C6_2_oh2_L | TGGGCCCAATTTTCCGTGACAATTAATTTCAGARCARATYGTGATGAA | 48 | |
| | | | | | | 6_B_R | C6_2_oh2_R | RCCTAATAAAGTGACYGCYANNNNNNNNNNGAATGAGTGTGCGTGCACTC | 50 | Phos. |
| 7 | 53 | ACT/ MIR | C | ACT-1-10/12-15/17-25/27-39 and MIR-1-18 | Cephalosporins | 7_A_L | C7_2_oh2_L | TGGGCCCAATTTTCCGTGACAATTAATTARCCHCTYARNCTGRACCAT | 48 | |
| | | | | | | 7_A_R | C7_2_oh2_R | ACHTGGATTAACGTBCCSAANNNNNNNNNNGAATGAGTGTGCGTGCACTC | 50 | Phos. |
| | | | | | | 7_B_L | C7_3_oh2_L | TGGGCCCAATTTTCCGTGACAATTAATTAAGMRBMRCATTWCGCCWGG | 48 | |
| | | | | | | 7_B_R | C7_3_oh2_R | GGVTAYCGYGABGGTAARGCNNNNNNNNNNGAATGAGTGTGCGTGCACTC | 50 | Phos. |
| 8 | 53 | CTX-M group 9 | A | CTX-M-9/13/14/16/17/19/21/24/27/ 38/46-51/64/65/67/73/81/83-87/90/ 93/98/ 99/102/104/105/110-113/ 121-123/125/126/129/130/132/ 134/137/147/148/159/161/168/174 | Extended-spectrum cephalosporins | 8_A_L | C8_2_oh2_L | TGGGCCCAATTTTCCGTGACAATTAATTGRAGGCGTGACGGCTTTTGC | 48 | |
| | | | | | | 8_A_R | C8_2_oh2_R | CCGCKMGATCGGCGATGAGANNNNNNNNNNGAATGAGTGTGCGTGCACTC | 50 | Phos. |
| | | | | | | 8_B_L | C8_3_oh2_L | TGGGCCCAATTTTCCGTGACAATTAATTCGTCTGGATCGCACTGAABV | 48 | |
| | | | | | | 8_B_R | C8_3_oh2_R | TACGCWGAATACCGCCATTCNNNNNNNNNNGAATGAGTGTGCGTGCACTC | 50 | Phos. |
| 9 | 52 | IMP | B | IMP-1-35/37/38/40-45/48/49/51-56/58 | Carbapenems | 9_A_L | C9_2b_oh2_L | TGGGCCCAATTTTCCGTGACAATTAATTTTTYCATRGYGAYAGYDCRG | 48 | |
| | | | | | | 9_A_R | C9_2b_oh2_R | SNGGAATWGRRTGGCTTAAYNNNNNNNNNNGAATGAGTGTGCGTGCACTC | 50 | Phos. |
| | | | | | | 9_B_L | C9_3b_oh2_L | TGGGCCCAATTTTCCGTGACAATTAATTGGAATWGRRTGGCTTAAYTC | 48 | |
| | | | | | | 9_B_R | C9_3b_oh2_R | TCRRTCVATYYCMACRTATGNNNNNNNNNNGAATGAGTGTGCGTGCACTC | 50 | Phos. |

| Cluster | No. of genes | Enzyme family | Classa | Genes targeted | Distinctive substrates | Probe Set | Oligo Name | Sequence(5'-3') | Length (bp) | 5' Mod |
|---|---|---|---|---|---|---|---|---|---|---|
| 10 | 48 | VIM | B | VIM-1-20/23-47/49-51 | Carbapenems | 10_A_L | C10_1_oh2_L | TGGGCCCAATTTTCCGTGACAATTAATTCGAARAACACRGYRGCMCTT | 48 | |
| | | | | | | 10_A_R | C10_1_oh2_R | CTCGCGGAGATTGARAAGCANNNNNNNNNNGAATGAGTGTGCGTGCACTC | 50 | Phos. |
| | | | | | | 10_B_L | C10_2_oh2_L | TGGGCCCAATTTTCCGTGACAATTAATTCGCACTTYCATGACGAYCGM | 48 | |
| | | | | | | 10_B_R | C10_2_oh2_R | GTCGGYGGMGTTGATGYCCTNNNNNNNNNNGAATGAGTGTGCGTGCACTC | 50 | Phos. |
| 12 | 38 | OXA (OXA-213 family) | D | OXA-213/267-273/304/305/322-332/348-354/357-359/417/421/500-503/506 | Carbapenems | 12_A_L | C12_2b_oh2_L | TGGGCCCAATTTTCCGTGACAATTAATTAYGGHCARAARCGYTTRTTT | 48 | |
| | | | | | | 12_A_R | C12_2b_oh2_R | CCYGAYTGGRAAAARGAYATNNNNNNNNNNGAATGAGTGTGCGTGCACTC | 50 | Phos. |
| | | | | | | 12_B_L | C12_5b_oh2_L | TGGGCCCAATTTTCCGTGACAATTAATTTWACVGGYTGGRTNRTTCAA | 48 | |
| | | | | | | 12_B_R | C12_5b_oh2_R | YMDCARGGARAAATTRTHGCNNNNNNNNNNGAATGAGTGTGCGTGCACTC | 50 | Phos. |
| 13 | 30 | GES | A | GES-1-22/24-31 | Cephalosporins, extended-spectrum cephalosporins, carbapenems | 13_A_L | C13_1_oh2_L | TGGGCCCAATTTTCCGTGACAATTAATTAGCAGCTSAGATCGGTGTTG | 48 | |
| | | | | | | 13_A_R | C13_1_oh2_R | CGATCGTCGATCCCCAAGGANNNNNNNNNNGAATGAGTGTGCGTGCACTC | 50 | Phos. |
| | | | | | | 13_B_L | C13_3_oh2_L | TGGGCCCAATTTTCCGTGACAATTAATTGCCTCTGTCGGTCAAGTTAT | 48 | |
| | | | | | | 13_B_R | C13_3_oh2_R | TACACAACTCATCCTGAGCANNNNNNNNNNGAATGAGTGTGCGTGCACTC | 50 | Phos. |
| 14 | 30 | CMY/FOX/MOX | C | CMY-1/8/8b/9-11/19, FOX-1-10/12/13 and MOX-1-12 | Cephalosporins (CMY-8/9 have extended-spectrum) | 14_A_L | C14_2_oh2_L | TGGGCCCAATTTTCCGTGACAATTAATTGTCARYGAGCAGACSCTGTT | 48 | |
| | | | | | | 14_A_R | C14_2_oh2_R | CGAKATWGGVTCSGTSAGCANNNNNNNNNNGAATGAGTGTGCGTGCACTC | 50 | Phos. |
| | | | | | | 14_B_L | C14_3_oh2_L | TGGGCCCAATTTTCCGTGACAATTAATTGATVGSCRTCGTCATGCTGG | 48 | |
| | | | | | | 14_B_R | C14_3_oh2_R | CCAAYCGCAACTMYCCYAWCNNNNNNNNNNGAATGAGTGTGCGTGCACTC | 50 | Phos. |
| 15 | 27 | CARB | A | CARB-17-20/22/24-36/38/40/41/43-48 | Carbenicillin | 15_A_L | C15_1_oh2_L | TGGGCCCAATTTTCCGTGACAATTAATTAAGTTATTCCTGTTGGYTGG | 48 | |
| | | | | | | 15_A_R | C15_1_oh2_R | GCTGATGGTTTRCTCAACTGNNNNNNNNNNGAATGAGTGTGCGTGCACTC | 50 | Phos. |
| | | | | | | 15_B_L | C15_2_oh2_L | TGGGCCCAATTTTCCGTGACAATTAATTACCATGCTAAGCGAYATGGA | 48 | |
| | | | | | | 15_B_R | C15_2_oh2_R | BAGCGGCAAACTCAACAAAANNNNNNNNNNGAATGAGTGTGCGTGCACTC | 50 | Phos. |
| 18 | 22 | CTX-M group 2 | A | CTX-M-2/4-7/20/21/35/43/44/56/59/76/77/92/95/97/115/124/131/141/165 | Extended-spectrum cephalosporins | 18_A_L | C26_18L | TGGGCCCAATTTTCCGTGACAATTAATTCAATGTTAACGGTGATGGCG | 48 | |
| | | | | | | 18_A_R | C26_18R | ACGCTACCCCTGCTATTTAGNNNNNNNNNNGAATGAGTGTGCGTGCACTC | 50 | Phos. |
| | | | | | | 18_B_L | C105_18L | TGGGCCCAATTTTCCGTGACAATTAATTGCAGCTGRAAGCCCTGGAGA | 48 | |
| | | | | | | 18_B_R | C105_18R | AAAGTTCGGGAGGTCGGCTTNNNNNNNNNNGAATGAGTGTGCGTGCACTC | 50 | Phos. |
| 20 | 20 | OXA (OXA-48 family) | D | OXA-48/54/162/163/181/199/204/232/244/245/247/252/370/405/416/436/438/439/484/505 | Carbapenems | 20_A_L | C1_20L | TGGGCCCAATTTTCCGTGACAATTAATTATGCGTGYRTTAGCCTTATC | 48 | |
| | | | | | | 20_A_R | C1_20R | GRCTGTRTTDDTGGTGRCAWNNNNNNNNNNGAATGAGTGTGCGTGCACTC | 50 | Phos. |
| | | | | | | 20_B_L | C146_20L | TGGGCCCAATTTTCCGTGACAATTAATTAGAAYAMRCAGCARGGWTTT | 48 | |
| | | | | | | 20_B_R | C146_20R | ACCAAYRATCTTAARCGGGCNNNNNNNNNNGAATGAGTGTGCGTGCACTC | 50 | Phos. |
| 27 | 14 | CTX-M group 8 and group 25 | A | CTX-M-8/25/26/39-41/63/78/89/91/94/100/152/160 | Extended-spectrum cephalosporins | 27_A_L | C393_27L | TGGGCCCAATTTTCCGTGACAATTAATTGTACAGCGAYAATACKGCCA | 48 | |
| | | | | | | 27_A_R | C393_27R | TGAAYAAGCTGATTGCCCATNNNNNNNNNNGAATGAGTGTGCGTGCACTC | 50 | Phos. |
| | | | | | | 27_B_L | C584_27L | TGGGCCCAATTTTCCGTGACAATTAATTATCTSACRTTGGGCARTGCC | 48 | |
| | | | | | | 27_B_R | C584_27R | YTRGGTGAMACTCAGCGTGCNNNNNNNNNNGAATGAGTGTGCGTGCACTC | 50 | Phos. |
