# Supplementary material for: Digital multiplex ligation assay for highly multiplexed screening of β-lactamase-encoding genes in bacterial isolates
Source: Commun Biol. 2020 May 25;3:264. doi: 10.1038/s42003-020-0980-7 (PMC7248093; doi:10.1038/s42003-020-0980-7)
Supplement: Supplementary file 2 — Reporting Summary [file 42003_2020_980_MOESM2_ESM.pdf]

nature research

Corresponding author(s): DBPR COMMSBIO-19-1511

Last updated by author(s): Mar 16, 2020

Reporting Summary

Nature Research wishes to improve the reproducibility of the work that we publish. This form provides structure for consistency and transparency in reporting. For further information on Nature Research policies, see Authors & Referees and the Editorial Policy Checklist .

Statistics

For all statistical analyses, confirm that the following items are present in the figure legend, table legend, main text, or Methods section.

n/a	Confirmed	
☐	☒	The exact sample size (*n*) for each experimental group/condition, given as a discrete number and unit of measurement
☐	☒	A statement on whether measurements were taken from distinct samples or whether the same sample was measured repeatedly
☒	☐	The statistical test(s) used AND whether they are one- or two-sided<br>*Only common tests should be described solely by name; describe more complex techniques in the Methods section.*
☒	☐	A description of all covariates tested
☒	☐	A description of any assumptions or corrections, such as tests of normality and adjustment for multiple comparisons
☒	☐	A full description of the statistical parameters including central tendency (e.g. means) or other basic estimates (e.g. regression coefficient) AND variation (e.g. standard deviation) or associated estimates of uncertainty (e.g. confidence intervals)
☒	☐	For null hypothesis testing, the test statistic (e.g. *F*, *t*, *r*) with confidence intervals, effect sizes, degrees of freedom and *P* value noted<br>*Give P values as exact values whenever suitable.*
☒	☐	For Bayesian analysis, information on the choice of priors and Markov chain Monte Carlo settings
☒	☐	For hierarchical and complex designs, identification of the appropriate level for tests and full reporting of outcomes
☒	☐	Estimates of effect sizes (e.g. Cohen's *d*, Pearson's *r*), indicating how they were calculated

Our web collection on statistics for biologists contains articles on many of the points above.

Software and code

Policy information about availability of computer code

Data collection	Data were collected using software built-in Illumina HiSeq
Data analysis	Data were analyzed using a combination of custom code (dmla and epride, available at github https://github.com/manutamminen/dmla/ and https://github.com/manutamminen/epride/) and open source code (nsearch, described in Schmid et al. 2018)

For manuscripts utilizing custom algorithms or software that are central to the research but not yet described in published literature, software must be made available to editors/reviewers. We strongly encourage code deposition in a community repository (e.g. GitHub). See the Nature Research guidelines for submitting code & software for further information.

Data

Policy information about availability of data

All manuscripts must include a data availability statement. This statement should provide the following information, where applicable:

- Accession codes, unique identifiers, or web links for publicly available datasets
- A list of figures that have associated raw data
- A description of any restrictions on data availability

The sequence data (PRJNA531165) is available at SRA.

Field-specific reporting

Please select the one below that is the best fit for your research. If you are not sure, read the appropriate sections before making your selection.

☒ Life sciences ☐ Behavioural & social sciences ☐ Ecological, evolutionary & environmental sciences

Life sciences study design

All studies must disclose on these points even when the disclosure is negative.

Sample size	The number of isolates tested (74) was based on the availability of a 60 strain isolate library and 14 environmental isolates.
Data exclusions	The data from 18 clusters is reported in the manuscript. Each cluster contains two probe pairs. The data from a 19th cluster was excluded because the cluster only contained one probe pair, and the algorithm for calculating presence/absence of a gene within a bacterial isolate is based on the combined probability of two probe pairs.
Replication	The study was replicated with two sequencing runs, one with 35 PCR cycles (reported in the paper) and one with 40 PCR cycles (not reported in the paper). The results are identifical, but were not reported in the paper as the number of cycles was different.
Randomization	The experiment does not require randomization of samples.
Blinding	There is no group assignment, and so there was no blinding.

Reporting for specific materials, systems and methods

We require information from authors about some types of materials, experimental systems and methods used in many studies. Here, indicate whether each material, system or method listed is relevant to your study. If you are not sure if a list item applies to your research, read the appropriate section before selecting a response.

Materials & experimental systems

n/a	Involved in the study
☒	☐ Antibodies
☒	☐ Eukaryotic cell lines
☒	☐ Palaeontology
☒	☐ Animals and other organisms
☒	☐ Human research participants
☒	☐ Clinical data

Methods

n/a	Involved in the study
☒	☐ ChIP-seq
☒	☐ Flow cytometry
☒	☐ MRI-based neuroimaging
